# Le professeur Michel Rey est décédé le 2 Avril 2022

**DOI:** 10.48327/mtsi.v2i4.2022.283

**Published:** 2022-11-04

**Authors:** Jean BEYTOUT

**Affiliations:** CHU Clermont-Ferrand, Site Gabriel-Montpied, 58 rue Montalembert, 63000 Clermont-Ferrand, France; Photos publiées avec l'aimable autorisation de Madame Marie-Anne Cuillé, fille du Professeur Michel Rey

Michel Rey est né le 5 mai 1931 à Lyon. Il effectue ses études de médecine et son internat à Paris, notamment à l'hôpital Claude Bernard où il acquiert sa qualification en Pathologie infectieuse. Après avoir présenté sa thèse en 1954, il souhaite faire son service militaire en Afrique et il est affecté à Thiès (Sénégal) puis à Gao (Soudan français, aujourd'hui Mali) jusqu'en 1957. Après avoir poursuivi ses activités médicales et universitaires à Paris, il revient au Sénégal en 1961 où il est nommé chef du service des maladies infectieuses à l'hôpital Fann de Dakar. Il y acquiert une grande expérience des maladies infectieuses tropicales et s'intéresse aux problèmes de santé publique en Afrique de l'Ouest. Il multiplie les échanges avec les acteurs de santé, africains ou coopérants intervenant dans cette région du monde. Il développe une expertise particulière sur le tétanos, la poliomyélite, la rougeole et les maladies à prévention vaccinale: sa promotion de la vaccination contre la rougeole a un impact sanitaire remarquable et sa compétence est reconnue notamment par l'OMS.

**Figure 1:**
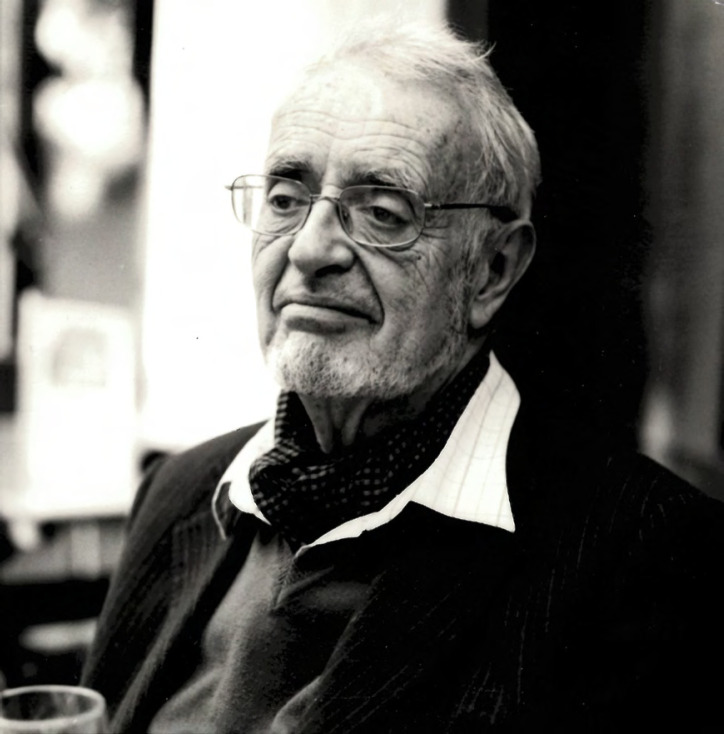
Michel Rey photographié par sa petite fille Laura Cuillé Michel Rey photographed by his granddaughter Laura Cuillé

De retour en France en 1971, il est nommé professeur à la faculté de médecine de Clermont-Ferrand et praticien hospitalier au Centre hospitalier universitaire. Il crée le service des maladies infectieuses et tropicales (1980) et met en place le Centre régional des vaccinations internationales et de conseil aux voyageurs. Outre une activité hospitalo-universitaire régionale intense, il est reconnu en France et dans le monde pour son expertise dans la prévention des maladies infectieuses, notamment par la vaccination: il crée la Ligue pour la Prévention des Maladies infectieuses et il est sollicité pour présider le Conseil supérieur d'hygiène publique de France. Il développe et entretient des relations étroites avec de nombreux correspondants africains et internationaux. Il est recruté comme expert à Genève dans la branche Maladies transmissibles de l'OMS où il exerce en 1985 et 1986. Puis il reprend la chefferie du service des maladies infectieuses de Clermont-Ferrand, poursuivant le développement de la spécialité jusqu'en 1994.

Retiré à Paris, professeur honoraire de la faculté de Clermont, il continue à avoir une importante activité: il participe aux activités des sociétés savantes françaises de pathologie exotique, de maladies infectieuses, d’épidémiologie et de prévention. Il est membre correspondant de l'Académie de Médecine depuis 1999. Il préside la Société (française) de Médecine des voyages entre 1994 et 2003 et accède même à la présidence de l'International Society of Travel Medicine (1997-1999). Depuis une dizaine d'années, des ennuis de santé l'ont conduit à renoncer à ses nombreuses activités…

Michel Rey laissera le souvenir d'un humaniste de grande culture et d'une très grande ouverture d'esprit. Il était d'une insatiable curiosité pour tout ce qui concernait ses contemporains, quelles que soient leurs origines ou leurs conditions, et il se sentait concerné par tout ce qui pouvait affecter leur vie. D'une grande convivialité, il avait le don de mettre à l'aise ses interlocuteurs, de les inciter à la réflexion plutôt que d'imposer ses idées. Ses connaissances s'appuyaient sur une large pratique de terrain, l'intégration raisonnée de l'expérience collective et le recours aux méthodes d'information les plus modernes disponibles. Dans son activitémédicale, outre sa compétence de spécialiste expérimenté, ses patients appréciaient combien il se souciait de leur bien-être. Ses collaborateurs reconnaissaient sa compétence et son autorité naturelle, mais aussi sa simplicité, et la facilité des relations qu'il instaurait autour de lui. Ses enseignements étaient particulièrement vivants et entraînants. Sa production scientifique, dans ses domaines de prédilection (prévention, vaccination), a été particulièrement riche et le plus souvent orientée vers l'application au plus grand nombre dans un objectif de santé publique. Il était très apprécié de ses collègues et a formé de nombreux élèves à l’étranger (notamment dans plusieurs pays d'Afrique) comme en France (tels les professeurs Jean Beytout et Henri Laurichesse qui lui ont succédé à la chefferie du service des maladies infectieuses et tropicales du CHU de Clermont-Ferrand). Il a réuni et organisé de nombreux congrès et séminaires dans plus de 20 pays, sur tous les continents. Michel Rey, dont l’épouse est décédée en 2000, avait 4 enfants, de nombreux petits-enfants et des arrière-petits-enfants. À toute sa famille et à ses proches, nous transmettons toutes nos condoléances et l'assurance que l'on peut garder de Michel Rey la mémoire d'un homme estimé et aimé de tous ceux qui l'ont rencontré.

**Figure 2:**
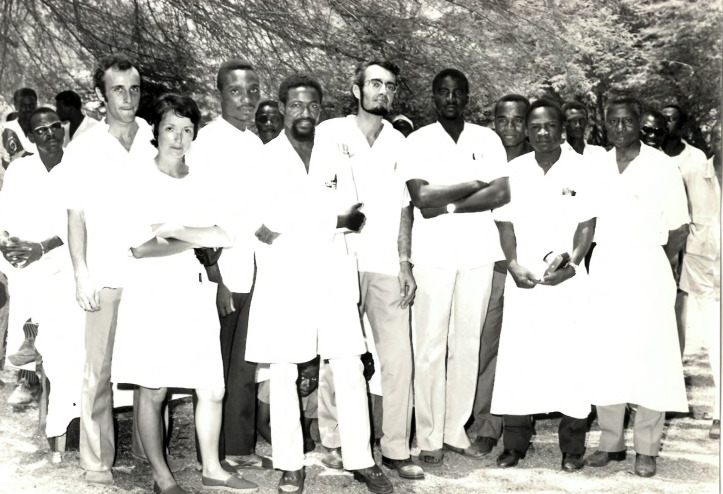
Michel Rey en Afrique (merci de vous faire connaître auprès de MTSI si vous reconnaissez des personnes sur cette photo) Michel Rey in Africa (please contact MTSI if you recognise anyone in this photo)

